# Molecular Dynamic Study on the Structure and Thermal Stability of Mutant Pediocin PA-1 Peptides Engineered with Cysteine Substitutions

**DOI:** 10.1007/s12602-024-10225-3

**Published:** 2024-03-01

**Authors:** Büşra SEVİM, Evrim GÜNEŞ ALTUNTAŞ

**Affiliations:** Ankara University Biotechnology Institute, Ankara, Turkey

**Keywords:** Pediocin PA-1 variants, Thermal stability, Disulfide bonds, Molecular dynamic simulations, Amino acid substitutions, Protein engineering

## Abstract

Pediocin and analogous bacteriocins, valued for thermal stability, serve as versatile antimicrobials in the food sector. Improving their resilience at high temperatures and deriving derivatives not only benefit food production but also offer broad-spectrum antimicrobial potential in pharmaceuticals, spanning treatments for peptic ulcers, women’s health, and novel anticancer agents. The study aims to create mutant peptides capable of establishing a third disulfide bond or enhanced through cysteine substitutions. This involves introducing additional Cys residues into the inherent structure of pediocin PA-1 to facilitate disulfide bond formation. Five mutants (Mut 1–5) were systematically generated with double Cys substitutions and assessed for thermal stability through MD simulations across temperatures (298–394 K). The most robust mutants (Mut 1, Mut 4–5) underwent extended analysis via MD simulations, comparing their structural stability, secondary structure, and surface accessibility to the reference Pediocin PA-1 molecule. This comprehensive assessment aims to understand how Cys substitutions influence disulfide bonds and the overall thermal stability of the mutant peptides. In silico analysis indicated that Mut 1 and Mut 5, along with the reference structure, lose their helical structure and one natural disulfide bond at high temperatures, and may impacting antimicrobial activity. Conversely, Mut 4 retained its helical structure and exhibited thermal stability similar to Pediocin PA-1. Pending further experimental validation, this study implies Mut 4 may have high stability and exceptional resistance to high temperatures, potentially serving as an effective antimicrobial alternative.

## Introduction

Bacteriocins, synthesized ribosomally by various bacterial species, possess paramount importance as antimicrobial peptides due to their versatile nature. They demonstrate a wide spectrum of antimicrobial effects, varying from broad to narrow, capable of affecting numerous related or unrelated strains because of these inherent attributes [1–7]. In recent years, bacteriocins have garnered increasing significance, primarily due to their versatile applicability in both the health and food industries. Bacteriocins find application in various domains, including harnessing their probiotic properties within the pharmaceutical industry, addressing pathogen-induced illnesses, managing conditions like peptic ulcers, contributing to cancer therapy, and supporting women’s health [6, 7]. The demand for bacteriocins, particularly in the food sector, stems from the growing need for pathogen-free, naturally sourced products with fewer chemical preservatives [1, 5, 8, 9]. These antimicrobial peptides have proven to be exceptional food preservatives within the food industry. Some key attributes that underscore their importance include their resistance to pH variations and heat, their ability to inhibit foodborne pathogens even at low concentrations, and their susceptibility to deactivation by intestinal proteases [9]. Additionally, in a world increasingly concerned about the emergence of antibiotic-resistant bacterial strains, the quest for antimicrobial alternatives to conventional antibiotics, particularly in the healthcare domain, amplifies the significance of bacteriocins [1, 2, 5, 10].

Bacteriocins are categorized into four main classes, where Class IV, known as “bacteriolysins,” contains complexes containing carbohydrate and lipid moieties. Beyond Class IV, bacteriocins are divided into three primary classes: Class I, characterized by post-translational modifications; Class II, which lacks these modifications or has a cyclic structure; and Class III, comprising heat-sensitive and large-sized bacteriocins [1, 11].

Pediocin and pediocin-like bacteriocins are classified within the Class IIa bacteriocins. The subject of this study, pediocin, distinguishes itself from other bacteriocins based on several key properties. Typically comprising 37–48 amino acids, pediocins demonstrate anti-listerial characteristics, exhibit resistance to heat (up to 121 °C), and possess peptide structures incorporating one or two disulfide bonds [10, 12, 13]. These bacteriocins are primarily produced by lactic acid bacteria (LAB), especially by pediococci and lactobacilli species [5]. They share a highly conserved peptide region known as the “pediocin box,” which is located in the highly conserved N-terminal region [1, 10, 12, 14–16]. The amino acid sequence in the C-terminal region of these bacteriocins may vary among different bacteriocin variants. Pediocin PA-1/AcH stands as the most well-known member of these bacteriocins, boasting a heat resistance of 80 °C and beyond. It is produced by LAB and ranks as one of the most extensively studied bacteriocins, following nisin [4, 16, 17].

Pediocin PA-1/AcH is characterized by a structural configuration featuring two crucial disulfide bonds, specifically Cys9-Cys14 and Cys24-Cys44. Extensive documentation highlights the pivotal role of the disulfide bond, particularly in the C-terminal region, in the antimicrobial efficacy of this bacteriocin [10, 12, 16, 18]. These disulfide bonds facilitate its antimicrobial activity by binding to the pore-forming receptor mannose phosphotransferase (Man-PTS) system [6]. The hypothesis of this study was formulated based on the premise that, in the process of pore formation using this way, the potential formation of the third disulfide bond, in addition to the second disulfide bond, may enhance the structural stability. Additionally, it is well-established that bacteriocins lacking disulfide bonds in this specific region demonstrate significantly reduced antimicrobial activity, exhibiting a 30–50 fold reduction at 37 °C compared to pediocin PA-1 [12]. Furthermore, the presence of the second disulfide bond within the C-terminal region acts as a robust protective mechanism, preserving the structural integrity of the peptide even under high-temperature conditions [15].

Pediocin exhibits lethal effects on *Listeria monocytogenes*, a pathogen causing infections in various food items. The disulfide bonds' contribution to heat resistance and structural stability highlights the substantial utility of pediocin in ensuring food safety. This is further supported by the correlation between temperature-sensitive antimicrobial activity and the optimal growth temperature of bacteria that produce pediocin. Moreover, given the current era characterized by growing antibiotic resistance as a significant health issue [19], technologically modified and improved versions of pediocin could potentially offer alternative therapeutic options to antibiotics across multiple domains.

The infusion of information technologies into fundamental scientific fields has driven the rising adoption of in silico methodologies, particularly molecular dynamics studies. These methodologies strive to uncover the structural characteristics of peptides and acquire data that could present hurdles in traditional wet lab experiments, especially in pharmaceutical research. Molecular dynamics (MD) studies, a subset of in silico methods, simulate atomic-level movements of molecules, conforming to biophysical principles under specified conditions [20].

MD studies are essential tools for investigating molecular conformational changes and ligand interactions, offering insights into diverse biological processes at the femtosecond timescale, sought after in research [20, 21]. Integrating MD simulations to improve bacteriocin variants, like pediocin, is pivotal for the food and pharmaceutical sectors. It reduces wet-lab costs, accelerates processes, and contributes to scholarly and industrial advancements, expediting publications.

The current study utilized MD simulations to explore the influence of amino acid substitutions in pediocin PA-1 on peptide stability across different temperature ranges. Considering the significance of eukaryotic defensins, which are cysteine-rich antimicrobial peptides pivotal for fortifying the innate immune system of host, and potential defensin-like structure in bacteriocins (e.g., class II bacteriocins such as bactofencin and laterosporuli10) [19, 22, 23], this study also assessed the feasibility of integrating a third disulfide bond into the structure of pediocin PA-1. To enhance structural stability and potential disulfide bond formation, introduced double Cys substitutions within the peptide. These residues have the capacity to reinforce the stability of the secondary structure in their free form and promote the formation of disulfide bonds. Subsequent to this, conducted MD simulations on these peptides across a temperature spectrum ranging from 298 to 394 K (equivalent to 25 to 121 °C). The objective was to investigate peptide thermal stability and evaluate the influence of replaced Cys residues in potentially aiding the formation of disulfide bonds. The focus of this research on peptide enhancement and engineering holds particular relevance within industrial environments, particularly those linked to higher temperatures. The creation of new pediocin variants tailored for industrial use introduces promising antimicrobial peptides with potential applications in addressing infectious diseases such as Listeriosis, diverse health-related purposes, and bolstering food safety protocols.

## Materials and Methods

### Obtaining the Three-Dimensional Structure of Pediocin PA-1 and Constructing Mutant Molecules

The three-dimensional structure of Pediocin PA-1 was obtained using the NMR data available in the Protein Data Bank (PDB) with the corresponding PDB code: 5UKZ, serving as the reference for 3D structure of pediocin. To construct mutant peptide structures, PyMOL version 2.5 [20] was employed, and details regarding the amino acid substitutions are provided in Table 1. Subsequently, an analysis of the potential effects of these mutations on the structure and functionality of the peptides was conducted. This analysis was facilitated by the HOPE server [21].

**Table 1:** Mutant pediocin constructs selected for thermostability analysis

**Name of Peptide**	**Region of Substitution**
**Mut 1**	Ser13 → Cys13 Gln39 → Cys39
**Mut 2**	His38 → Cys38 Gly40 → Cys40
**Mut 3**	Asp17 → Cys17 Lys20 → Cys20
**Mut 4**	Thr35 → Cys35 Gly37 → Cys37
**Mut 5**	Asn28 → Cys28 Gln39 → Cys39

### Model Validations

The stereochemical quality of the model was evaluated using PROCHECK and MolProbity programs, which quantified the residues within the permitted regions of the Ramachandran plot [24, 25]. To further ascertain the reliability and quality of the protein structure, QMEAN *z*-scores from the QMEAN server (https://swissmodel.expasy.org/qmean/) and ProSA-web server were employed [26, 27].

### MD Simulations

The structural minimization process employed GROMACS (version 2022.3) [28–30]. The protein preparation involved utilizing the all-atom optimized potentials for liquid simulations (OPLS-AA) force field [31, 32] utilized the transferable intermolecular potential with three points (TIP3P) water model [33, 34]. To solvate the protein, it was positioned at the center of a rhombic dodecahedron box, established as the basic unit cell in the NPT ensemble [35], at a distance of 1 Angstrom (Å) from the box edge. Chlorine ions (Cl^−^) were incorporated to neutralize the system and balance the net charge. The solvated and electroneutral system underwent energy minimization utilizing the steepest descent method. Following the minimization step, a 100-ps NVT (constant number of particles, volume, and temperature) equilibration phase was conducted to stabilize the system. This was succeeded by a 100-ps NPT equilibration phase aimed at stabilizing the pressure (1 atm using the periodic boundary condition (PBC)). MD simulations were executed utilizing the computational resources available at servers of TÜBİTAK ULAKBİM’s High Performance and Grid Computing Center (TRUBA), which integrated the GROMACS (version 2022.3) program [28–30]. These simulations were conducted at various temperatures (298, 310, 313, 323, 333, 343, 348, 353, 363, 373, and 394 K; equivalent to 25, 37, 40, 50, 60, 70, 75, 80, 90, 100, and 121 °C) to acquire initial data and refine the structures. Initial MD simulation of the peptides involved 100 ns of constant pressure equilibration without constraints to relax the system. Subsequently, structures demonstrating stability, as confirmed by Root Mean Square Deviations (RMSD), Root Mean Square Fluctuations (RMSF) (all of these data were used for evaluation of changes in structure and dynamics of the mutant peptides), and data regarding the secondary structure of proteins (DSSP), were selected for further MD simulations, extending the primary simulation duration to 300 ns.

Analysis of simulation data utilized VMD (Visual Molecular Dynamics) (version 1.9.2) [36] and PyMOL (version 2.5) [37] for visualizing molecular structures. Graphical representations of RMSD, RMSF, DSSP, and Solvent Accessible Surface Area (SASA) were generated and interpreted during the examination of MD simulation results.

## Results and Discussion

### Evaluation of the Physicochemical Structure in HOPE Server and SASA Analysis After MD Simulation of the Mutant Peptides

**Fig. 1:**
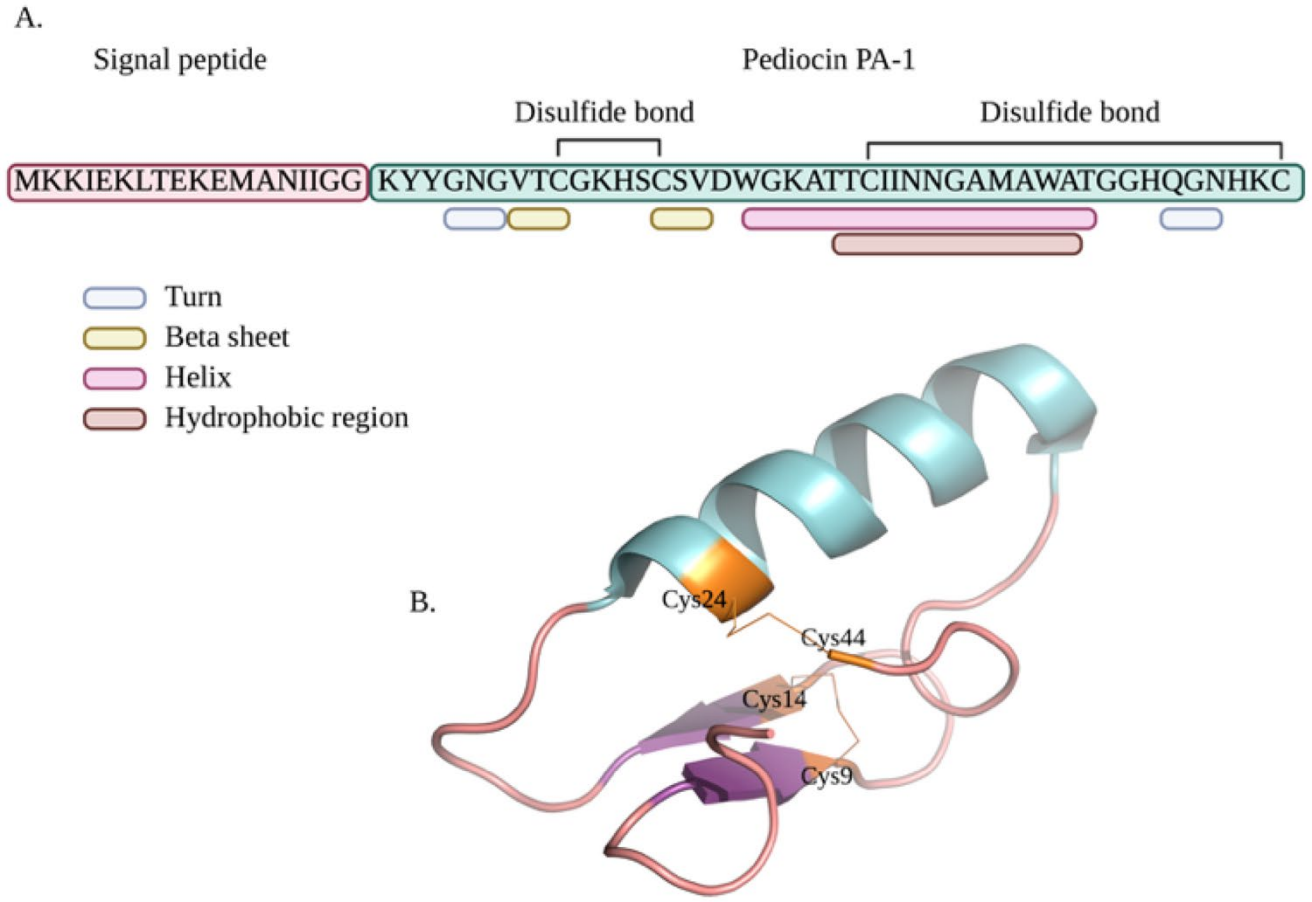
The 3D structure of the Pediocin PA-1 molecule (PDB Code: 5UKZ) was chosen as the reference structure. The blue region represents the α-helix, and the magenta arrows represent the β-sheet regions (Figure created with the BioRender app)

The pediocin molecule initially exists in a precursor form known as “pre-pediocin,” where it incorporates a signal peptide comprising 62 amino acids (as depicted in Fig. 1A). Notably, pre-pediocin remains inactive and lacks the critical disulfide bonds between Cys-Cys necessary for its bactericidal activity. Activation occurs through the cleavage of the 19-amino acid signal peptide in the pre-pediocin molecule, a process catalyzed by proteases within the cytoplasm. This cleavage results in the transformation of the remaining 44 amino acid peptide structure into an exponentially active state. Within the active peptide, specific amino acid segments play pivotal roles in structuring its functionality. Amino acids located at positions 4–6 (GNG) and 39–41 (QGN) within the active peptide adopt a “turn” conformation. Additionally, amino acids positioned at 7–9 (VTC) and 14–16 (CSV) contribute to the formation of a “β-sheet” structure. Furthermore, the amino acids spanning the 18–35 region, recognized as the active site of pediocin, adopt a “α-helix” structure, thus shaping the peptide’s functional structure (as illustrated in Fig. 1A, B) [10].

In this study, we used the Pediocin PA-1 molecule (PDB Code: 5UKZ) as the reference structure in PyMOL, specifically to determine its effects on the α-helix structure of N-terminal region and to assess possible disulfide bonds. Amino acid substitutions were introduced to convert various residues into Cys residues. Free Cys residues in peptide structures that have not formed disulfide bonds can affect macromolecule activity and thermal stabilization. Additionally, free Cys residues that have not formed disulfide bonds can contribute to α-helix stabilization [38, 39]. Accordingly, we made five different mutations in the amino acid sequence of Pediocin PA-1. Information about these substitutions is presented in Table 1.

Model validation was conducted using MolProbity and PROCHECK servers for both the 5 mutant peptides generated and the reference Pediocin PA-1 structure. Table 2 includes *z*-scores and Ramachandran statistics obtained from two distinct tools: the ProSA web server [27] and QMEAN by SWISS-MODEL [26]. The *z*-score functions as an indicator of the overall quality of the model and is utilized to verify whether the *z*-score of input structures falls within the commonly observed range for native proteins of comparable sizes [27]. The Ramachandran plot serves as an assessment tool for the accuracy of predicted protein structures by forecasting their structural stereochemical characteristics. PROCHECK evaluates the overall model geometry, scrutinizing the residue-by-residue geometry, and provides an assessment of the stereochemical quality of the predicted model. Ideally, the desired outcome involves fewer than 2% of residues falling within the allowed region, with none residing in the disallowed or outlier regions [40, 41].

**Table 2:** Z-scores and Ramachandran plot values of reference and mutant peptide structures

	**ProSA web server z-score**	**QMEAN4 score**	**MolProbity z-score**	**Ramachandran percentages (PROCHECK)**	**Ramachandran percentages (MolProbity)**	**MolProbity Score**^a^
**Reference Peptid (PDB:5UKZ)**	-4.57	-0.02	0.69 ± 1.18	Core: 91.2% Allow: 8.8%	Favored: 97.62% Outliers:0.00%	0.99
**Mut1**	-5.45	-0.19	0.78 ± 1.16	Core: 91.2% Allow: 8.8%	Favored: 97.62% Outliers:0.00%	1.20
**Mut2**	-5.08	-1.36	-4.16 ± 0.78	Core: 82.9% Allow: 17.1%	Favored: 83.33% Outliers:0.00%	1.99
**Mut3**	-3.71	-2.26	-3.64 ± 0.81	Core: 88.2% Allow: 11.8%	Favored: 88.10% Outliers:0.00%	1.10
**Mut4**	-3.67	-1.26	-3.65 ± 0.83	Core: 88.6% Allow: 11.4%	Favored: 88.10% Outliers:0.00%	1.10
**Mut5**	-5.98	-0.97	-3.66 ± 0.85	Core: 88.2% Allow: 11.8%	Favored: 88.10% Outliers:0.00%	1.10

MolProbity score combines the clashscore, rotamer, and Ramachandran evaluations into a single score, normalized to be on the same scale as X-ray resolution

^a^100th percentile (N = 27,675, 0 Å—99 Å), 100th percentile is the best among structures of comparable resolution; 0th percentile is the worst

When considering the statistics from the Ramachandran plot and *z*-score data, all other mutant peptides derived from the reference structure exhibit statistically close values to one another. Evaluation of the *z*-score data and Ramachandran data, suitable for ProSA web server NMR structures, indicated that temperature and stability states were quantifiable in MD simulation based on these validation outcomes.

**Fig. 2:**
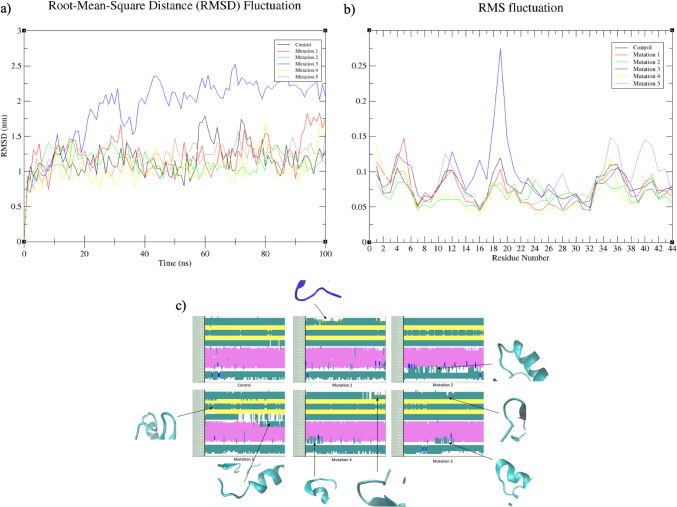
Evaluation of 5 mutations after 100 ns long MD simulation. **a** RMSD plot, **b** RMSF plot, and **c** DSSP analysis of mutant structures

It is noteworthy that structures displaying inadequate stabilization during the pre-screening phase were excluded from the study. Figure 2 illustrates the results of RMSD, RMSF, and DSSP analyses conducted during the 100 ns long MD simulation. Upon examining the RMSF and RMSD values, it becomes evident that Mut 3 exhibits considerable deviation. This outcome signifies a lower level of structural stability in Mut 3, notably observed in the RMSF graph, showcasing instability within the initial segment of the helical region. The stabilization of the β-bridge structure in Mut 3 is observed to be lost throughout most of the 100 ns MD simulation, particularly within the amino acid range of Cys17-Ala21, as depicted in Fig. 2c. The β-bridge has been linked to the functionality of peptide according to various researchers. Some researches noted a simultaneous decline in pediocin activity alongside reductions in β-strand and β-turn content [42], whereas another study noted underscored the significance of the β-strand in maintaining the well-balanced secondary structure of pediocin PA-1 [10]. In contrast, Mut 2 portrays a consistent profile in both the RMSF and RMSD graphs; however, the DSSP data indicates a potential loss of stabilization similar to that of Mut 3. Given the interaction of the α-helix structure with the Man-PTS system [6], the destabilization of the helical structure might influence this system. The stabilization of the helical structure may lead to disruptions within the Man-PTS system, presumed to be the mechanism behind the antimicrobial activity of pediocin, potentially causing a decline in receptor-pediocin affinity. Consequently, the loss of stability within the helical structure, particularly the reduction in temperature-related stabilization, could contribute to the decline in antimicrobial activity associated with the Man-PTS mechanism.

**Fig. 3:**
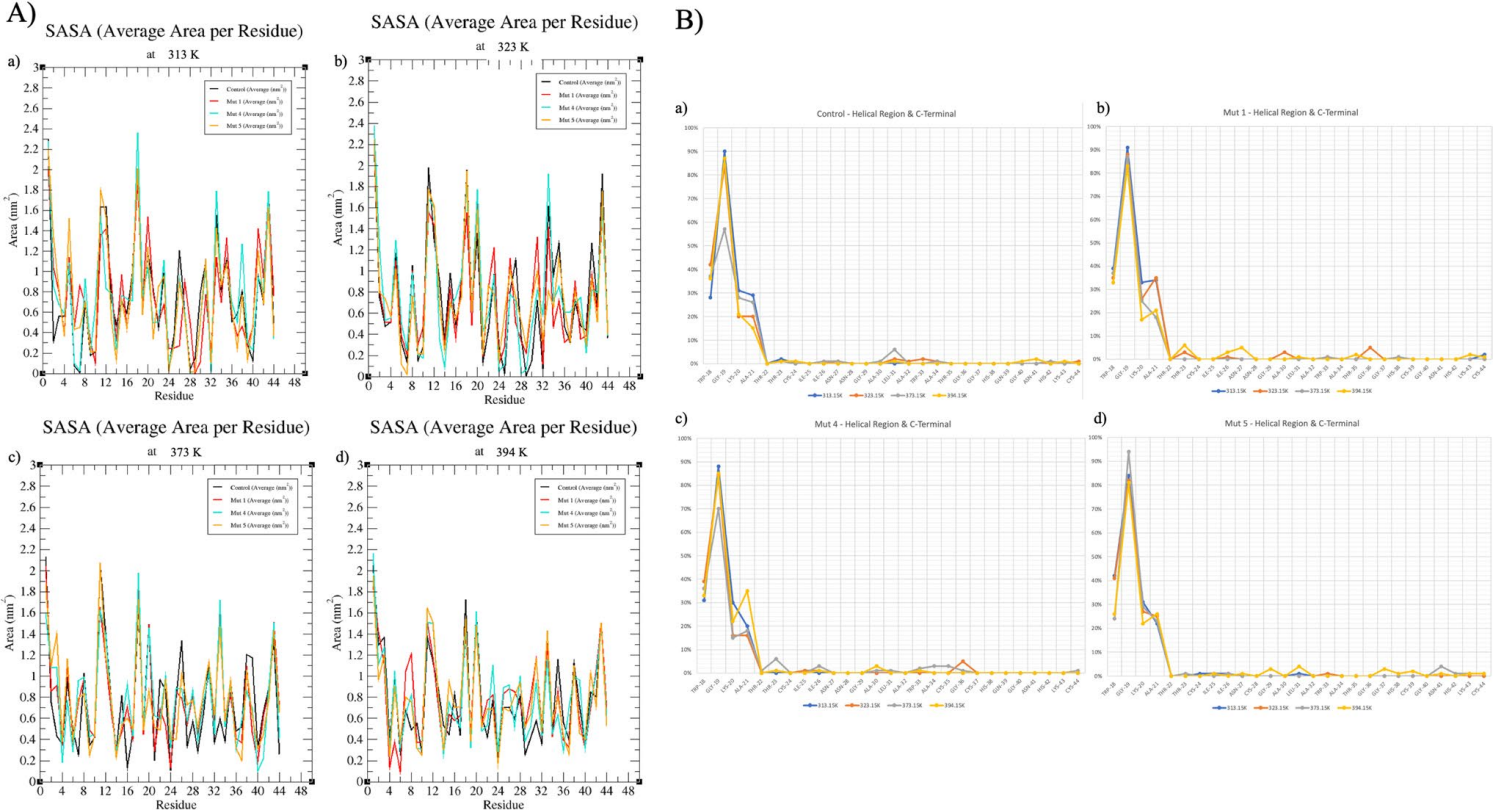
SASA values of reference peptide Pediocin PA-1 and the residues of mutations at different temperatures (**A**) and percentile expression of SASA values ranging from the helical region known to have an antimicrobial activity to the end of the C-terminal (**B**). **A** (a) 313 K, (b) 323 K, (c) 373 K, and (d) 394 K; **B** (a) reference, (b) Mut 1, (c) Mut 4, and (d) Mut 5

The anticipated impact of the introduced mutations on the protein encompasses both structural and functional aspects, as each amino acid carries unique properties relating to charge, size, polarity, and hydrophobicity. A comprehensive analysis of potential effects of these mutations on both structure and function was performed using the HOPE server [28], and the summarized outcomes are displayed in Table 3. Notably, among the implemented mutations, only Mut 4 exhibited an amino acid substitution that altered polarity; however, this specific mutation, particularly in terms of SASA (Solvent Accessible Surface Area) values (depicted in Fig. 3A, B), did not manifest any discernible changes.

**Table 3:** Possible effects of changing amino acids on the structure and function of the peptide

**Mutation**	**Region**	**Polarity**	**Charge**	**Hydrophobicity**	**Size of Residue**
**Mut 1**	S13C	Polar/ Not changed	Neutral/ Not changed	More Hydrophobic	Not changed
	Q39C	Polar/ Not changed	Neutral/ Not changed	More Hydrophobic	Decreased
**Mut 4**	T35C	Polar/ Not changed	Neutral/ Not changed	More Hydrophobic	Decreased
	G37C	Apolar → Polar	Neutral/ Not changed	More Hydrophobic	Increased
**Mut 5**	N28C	Polar/ Not changed	Neutral/ Not changed	More Hydrophobic	Decreased
	Q39C	Polar/ Not changed	Neutral/ Not changed	More Hydrophobic	Decreased

Different temperatures (313, 323, 373, and 394 K) were considered in computations to evaluate impact of disulfide bonds on stability of pediocin. Selections were based on factors like human body temperature and known thresholds for peptide degradation [43]. Bacteriocins, recognized as antimicrobials linked with probiotic microorganisms [14, 19, 44–48], usually exhibit effectiveness in the human intestinal environment. Therefore, temperature values were chosen considering their application in food environments and production as microbial metabolites.

The substitutions replaced the reference peptide with Cys amino acids, known for their comparatively greater hydrophobic nature than the original amino acids at the same positions within the peptide sequence. Despite the initial assumption that this substitution would enhance structural stability, a closer examination revealed that the Thr35 → Cys35 mutation in Mut 4 led to a 3% reduction in hydrophobicity at 373 K (100 °C) compared to the reference structure. Furthermore, this mutation induced a loss of hydrophobicity in neighboring amino acids. Similarly, Mut 5 exhibited a 2% decrease in hydrophobicity at 394 K (121 °C) due to the Gln39 → Cys39 mutation when contrasted with the reference peptide.

When examining the variations in SASA concerning residues in both the reference and mutant peptides across different temperatures, a consistent level of hydrophobicity is evident among all mutants within the temperature range of 313–323 K (40–50 °C). This constancy is notably conspicuous within the “TTCIINNGALAWA” residues, constituting the peptide’s hydrophobic region. At 313 K (40 °C) and 323 K (50 °C), the most structurally stable configurations are observed in the following sequence: the reference peptide, Mut 4, Mut 5, and Mut 1, respectively.

However, under higher temperatures (373–394 K or 100–121 °C), the SASA data (illustrated in Fig. 3) emphasize that, following the reference peptide, Mut 4 exhibits the highest degree of stabilization. The marginal fluctuations observed in the SASA graphs for Mut 4, along with these alterations closely resembling those in the reference peptide’s structure, particularly at 394 K (121 °C) concerning the critical antimicrobial site, strongly suggest that this mutant might demonstrate resistance in various industrial processes.

Glycine is notably more flexible compared to other amino acid residues, particularly concerning torsional angles, which significantly influences protein structural conformation [28]. However, the Gly37 → Cy37 substitution in Mut 4 was anticipated to reduce this inherent flexibility. Surprisingly, MD analysis demonstrated that this mutation enhances the helical structure's stability, especially at higher temperatures, indicating a potential stabilizing effect on the protein structure despite the expected decrease in glycine’s flexibility.

Another significant substitution, Asn28 → Cys28 in Mut 5, occurs within the hydrophobic region. Asparagine is less hydrophobic than Cysteine, implying that this mutation might promote stability within the hydrophobic region. Contrary to initial expectations, simulations revealed that Mut 5 exhibited the least stability among the mutants. This result underscores the intricate relationship between amino acid properties and overall structural dynamics, showcasing the complexity of protein behavior.

### Evaluation of MD Simulations in Terms of RMSD, RMSF, and DSSP

While Pediocin PA-1 has traditionally found application in the food industry, where it serves as a safeguard against pathogen contamination in fermented meat products, recent research suggests its potential utility in the milk and dairy products sector. Its stability in aqueous solutions, wide tolerance for pH variations, and minimal conformational changes when subjected to heating and freezing processes position pediocin as a versatile bacteriocin candidate with promising applications across the food industry [39, 45, 49]. In addition to its intended role in food preservation, there have been reports of potential of pediocin in cancer treatment [50]. According to studies in the scientific literature, it is believed that the hydrophobic region of the peptide could play a pivotal role in generating anti-carcinogenic effects. This is attributed to the hypothesis that increased hydrophobicity enhances the peptide's interaction with mammalian cell membranes, potentially leading to cytotoxic effects [51, 52].

MD simulations were conducted for both the reference molecule, Pediocin PA-1, and the mutant peptide molecules across a spectrum of temperatures, spanning from 298 to 394 K (equivalent to 25 to 121 °C). These simulations were extended over a duration of 300 ns. The evaluation of RMSD was employed to assess the stability alterations within these molecules throughout the simulation process. RMSD is an effective metric for gauging the extent of fluctuations exhibited by atoms within proteins concerning the reference structure. This metric proves especially advantageous in the context of thermostability assessments as it provides insights into the structural conformational changes experienced by macromolecules.

**Fig. 4:**
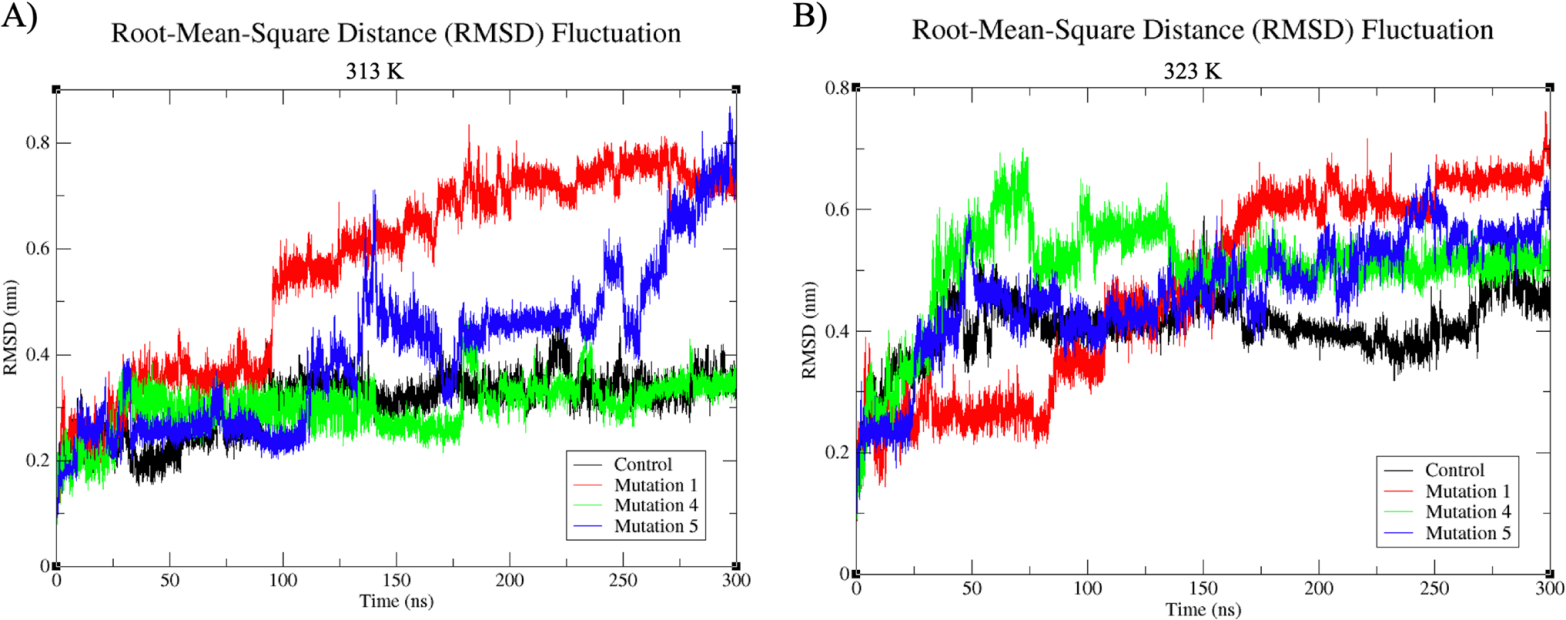
RMSD values of reference and mutant peptides at 313 K (40 °C) and 323 K (50 °C). **A** Thermostability of peptides at 313 K (40 °C). **B** Thermostability of peptides at 323 K (50 °C)

Class IIa bacteriocins, notably pediocin-producing variants, are recognized for their exceptional thermal stability, enduring temperatures as high as 121 °C [13]. To evaluate the thermal stability of both the original and mutant peptides within the 313 K to 323 K (40–50 °C) temperature range, RMSD plots derived from MD simulations were analyzed (Fig. 4). At 313 K (40 °C) (Fig. 4A), insights into the thermal stability of the reference and mutant peptide structures were obtained. Notably, the original molecule and the Mut 4 peptide structure showed similar deviations throughout the initial 85 ns of the simulations, maintaining structural stability akin to the reference structure [53]. Concurrent SASA data analysis supports this assessment, characterizing Mut 4 as a stable mutant (Fig. 3A, B). However, a distinct deviation arises with the Mut 1 peptide, which experiences an abrupt increase in deviation after 85 ns, reaching around 0.8 nm for the remaining simulation duration, indicating a significant loss of structural stabilization at 313 K (40 °C). Additionally, Mut 5 exhibits rapid deviation at 130 ns at 313 K (40 °C) and irregular fluctuations throughout the simulation, suggesting decreased stability under these conditions.

Figure 4B illustrates the thermal stability of both the reference and mutant peptides at 323 K (50 °C). During the initial 25 ns, all structures exhibit comparable deviations. Although, for the first 50 ns, the reference, Mut 4, and Mut 5 structures display parallel deviations, the distinctive fluctuation profile exhibited by Mut 4, particularly in the 75–150 ns range, stands out as noteworthy. This pattern implies that, at this temperature, Mut 4 exhibits relatively stable structural fluctuations, with a tendency toward consistency. This suggests that Mut 4 may experience reduced structural fluctuations and enhanced stabilization at this temperature.

However, despite the fluctuations observed at 313 K (40 °C), all structures, especially at 323 K (50 °C), maintain deviations within the range of 0.4–0.8 throughout the entirety of the simulation. This indicates that, at this higher temperature, all structures retain a relatively higher degree of stability, a crucial characteristic for peptides to preserve their antimicrobial activity.

**Fig. 5:**
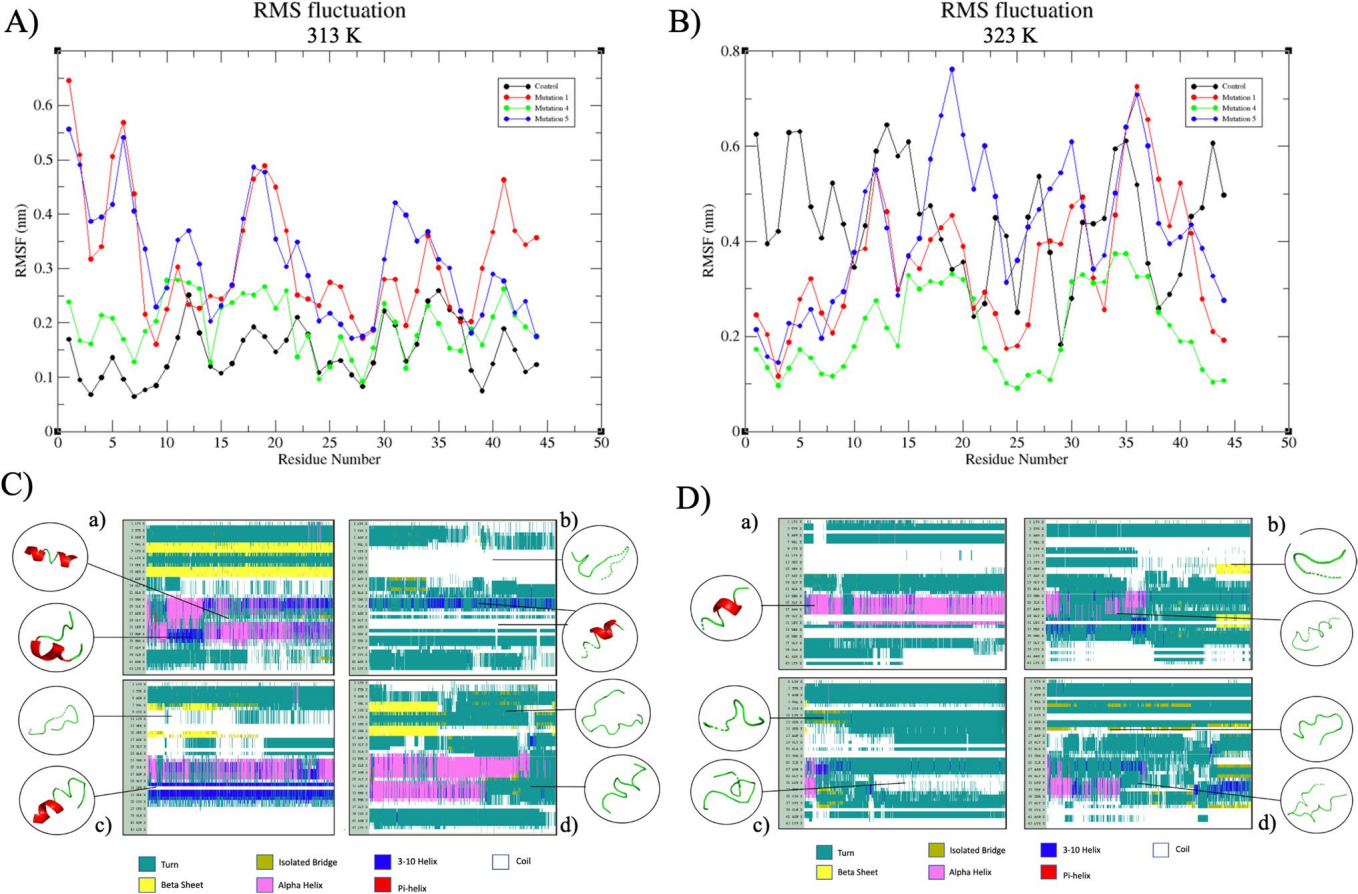
RMSF and DSSP evaluations of structures obtained as a result of MD simulations: **A** deviation data according to residues in the RMSF graph of structures at 313 K (40 °C); **B** deviation data of amino acids during simulation according to RMSF data at 323 K (50 °C); **C** DSSP analysis of peptides used in the study at 313 K (40 °C): (a) reference, (b) Mut 1, (c) Mut 4, and (d) Mut 5; **D** DSSP analysis of peptides used in the study at 323 K (50 °C): (a) reference, (b) Mut 1, (c) Mut 4, and (d) Mut 5

The analysis of RMSF and DSSP provides additional support for the investigation into thermal stability conducted through MD simulations. When examining the RMSF results of the simulations performed at both 313 K (40 °C) and 323 K (50 °C) (Fig. 5A, B), it becomes evident that Mut 1 and Mut 5 display pronounced fluctuations at 313 K (40 °C), particularly within the initial ten amino acid sequences (KYYGNGVTCG). This observation aligns with the RMSD and DSSP values. In accordance with the DSSP results, it is discerned that, at 313 K (40 °C), Mut 1 experiences heightened deviations in regions associated with the hydrophobic helical structure, known for its antimicrobial effects, and a well-protected area inclusive of the “β-sheet” (Fig. 5C-a). Additionally, the non-stable helical structure is prominently observed, further corroborated by SASA graphics. It becomes apparent that the α-helix structure cannot be maintained at this temperature and instead transitions into a 3^10^-helix structure. Given that the 3^10^-helix structure inherently represents an unstable helical conformation [54], this shift adversely impacts the stabilization of Mut 1, specifically within the peptide's active site, under these conditions. The RMSF and DSSP data collectively indicate that the active region of Mut 1 is not effectively shielded at 323 K (50 °C). Toward the end of the simulation, a secondary structure resembling a “β-sheet” structure emerges within the region previously occupied by the helical structure.

Similarly, Mut 5 exhibits a loss of its α-helix structure toward the latter part of the simulation, transitioning into a secondary structure profile primarily characterized by turn structures. The graph (Fig. 5) clearly illustrates that the substitution of Asn28 with Cys28 in Mut 5 triggers significant structural fluctuations commencing from Gly29, thereby contributing to destabilization within the peptide. It is reasonable to infer that Mut 1 and Mut 5 do not effectively preserve their helical structures when compared to the original peptide form. Consequently, their antimicrobial activities are expected to be notably diminished at both 313 K (40 °C) and 323 K (50 °C). Conversely, the helical regions within Mut 4 are presumed to remain relatively stable at 313 K (40 °C) when compared to the control, suggesting that its antimicrobial activity may approach or even match that of the reference peptide. However, this scenario is reversed for Mut 4 at 323 K (50 °C), where it exhibits a considerably less stable structural profile. The transformation of Gly37 to Cys37 renders Mut 4 more stable than the reference peptide structure, a conclusion supported by the SASA values derived from the simulations.

**Fig. 6:**
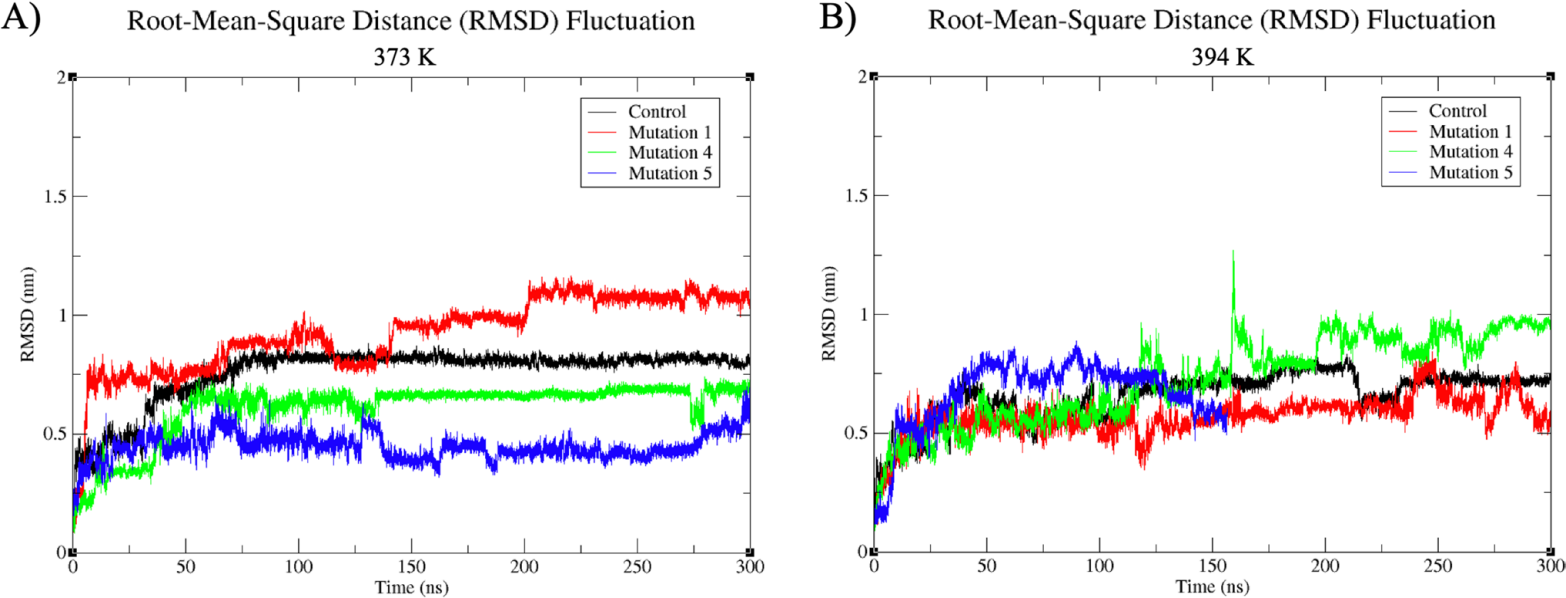
RMSD values showing the thermostability of reference and mutant peptides at 373 K and 394 K: **A** deviations at 373 K and **B** deviations at 394 K

To assess the antimicrobial potential of pediocin, especially in the context of the dairy industry, simulations were conducted at elevated temperatures, a scenario that it often encounters during sterilization processes and has been demonstrated to retain activity in various studies [35, 44, 55]. Figure 6 presents the RMSD data for the reference and mutant peptide structures at 373 K (100 °C) and 394 K (121 °C). Notably, the deviations observed in the peptide structures, typically within the range of 0.1–0.8 nm at 313 K (40 °C) and 323 K (50 °C), extend to the range of 0.2–1.25 nm at 373 K (100 °C). This indicates significantly reduced structural stabilization at these higher temperatures. In particular, Fig. 6A reveals that Mut 1 exhibits a tendency to stabilize within the 0.8–1.0 nm range after 10 ns, maintaining structural stability with the exception of a sharp fluctuation within the 120–125 ns range. Similarly, Mut 5, which initially demonstrates substantial fluctuations over the first 75 ns but subsequently settles within the 0.8–1.0 nm range, appears to maintain a relatively higher degree of stability at 373 K (100 °C) when compared to the reference and Mut 4 peptides. Upon examining the deviations in peptide structures at 394 K (121 °C), the highest temperature utilized in the MD simulation, it is observed that the structures tend to stabilize at levels ranging from 0.6–0.7 nm, beginning from 0.1 nm. Notably, while Mut 5 exhibits the most pronounced fluctuations at 394 K (121 °C), the reference structure, Pediocin PA-1, demonstrates the most stable structure with the least deviation. Consequently, it is advisable to investigate the activity of Mut 4, Mut 1, and Mut 5, respectively, under in vitro conditions to provide experimental validation and further insight into their potential [55].

**Fig. 7:**
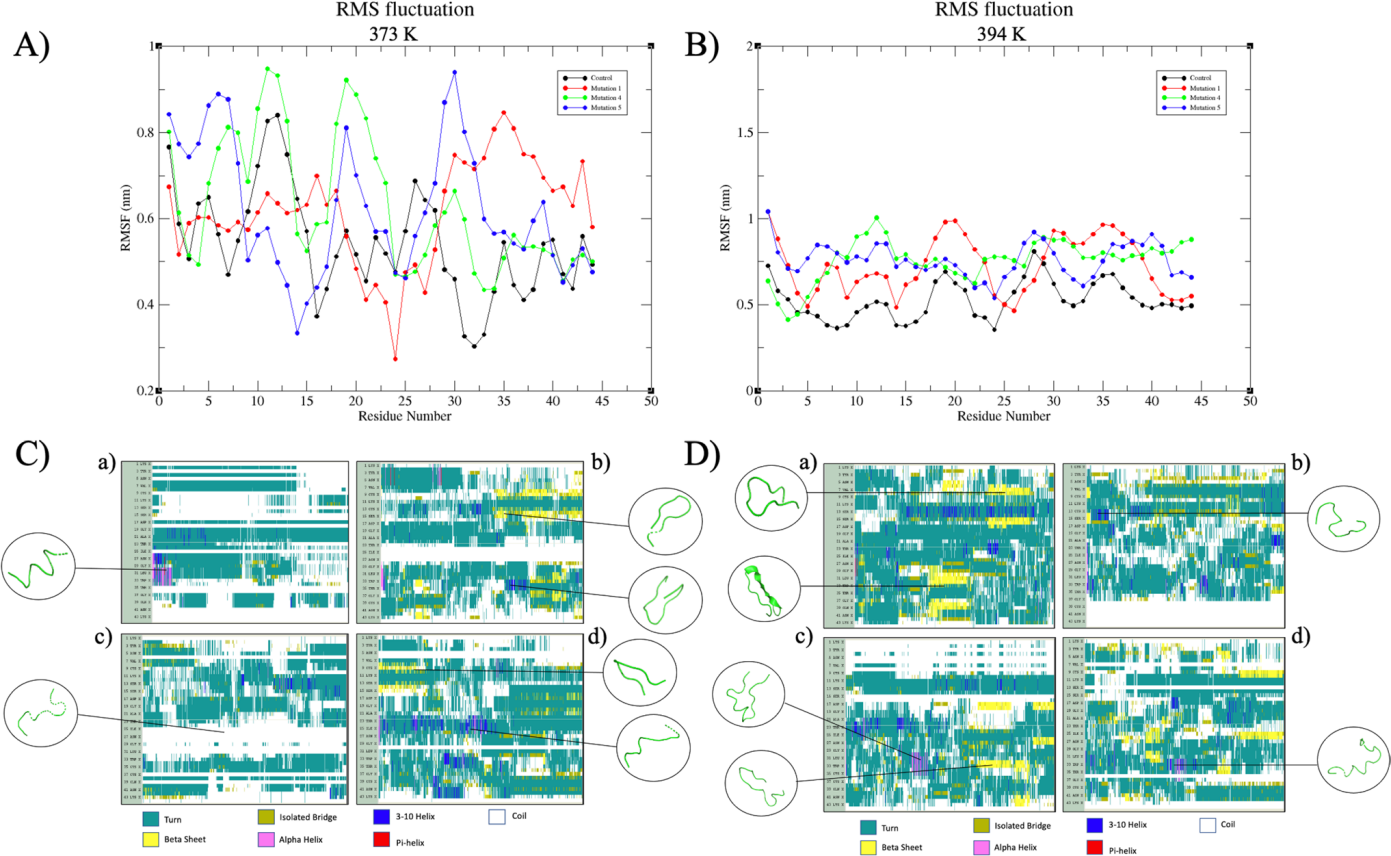
RMSF and DSSP evaluations of structures obtained as a result of MD simulations. **A** deviation data according to residues in the RMSF graph of structures at 373 K; **B** divergence data of amino acids during simulation according to RMSF data at 394 K; **C** DSSP analysis of peptides used in the study at 373 K: (a) reference, (b) Mut 1, (c) Mut 4, and (d) Mut 5; **D** DSSP analysis of peptides used in the study at 394 K: (a) reference, (b) Mut 1, (c) Mut 4, and (d) Mut 5

Figure 7 presents the RMSF and DSSP data obtained from MD simulations conducted at elevated temperatures. The RMSF values, which typically exhibited deviations within the range of 0.2–1 nm at 373 K (100 °C), expanded to 0.4–1.1 nm at 394 K (121 °C). At 373 K (100 °C), the DSSP data indicates that the reference peptide (Fig. 7C-a) has undergone nearly complete denaturation and has lost its significant secondary structure. Similarly, while the Mut 4 peptide has experienced a loss of secondary structure at 373 K (100 °C) (Fig. 7C-c), the β-sheet and 3^10^ helix structures, characterized by high deviations, were periodically observed in the structures of Mut 1 and Mut 5 throughout the simulation (Fig. 7C-b and d). However, these structures were not observed to be stable at 373 K (100 °C) due to their elevated deviation rates. At 394 K (121 °C), the DSSP data indicates that the reference structure exhibits a tendency to assume β-sheet structures; however, the RMSF values still suggest a high degree of instability. This suggests that while the reference structure tends to adopt a fixed conformation, it remains inherently unstable at this temperature. Despite the known resistance of bacteriocin class IIa peptides to temperatures ranging from 100 to 121 °C [13, 56], the relative instability of the pediocin PA-1 structure, the control structure, at 394 K (121 °C) raises questions regarding its antimicrobial effectiveness at such elevated temperatures. Notably, Mut 4 is a peptide structure in which the secondary structure in the region responsible for antimicrobial activity appears inclined toward renaturation. Although a helical structure is momentarily observed in this region during the simulation, DSSP analyses predominantly indicate a tendency to form β-sheet structures. Conversely, Mut 1 and Mut 5 fail to establish a clear secondary structure pattern at 394 K (121 °C) due to excessive deviations in both RMSF and DSSP analyses. Nonetheless, residues predisposed to β-sheet formation continue to be observed. Collectively, all data obtained at temperatures of 373 K (100 °C) and 394 K (121 °C) suggest that Mut 4 is a peptide that may undergo a more stable renaturation process following structural disruption when compared to the control structure. Consequently, it is advisable to investigate the in vitro antimicrobial effects of Mut 4.

### Evaluation of MD Analysis in Terms of Disulfide Bonds and Antimicrobial Activity Potential

The mechanism through which pediocin exerts its antimicrobial activity involves a hydrophilic structure, where the β-sheet region plays a critical role in integrating the hydrophobic α-helix structure responsible for antimicrobial activity. This integration acts as a hinge within the lipid layer of the plasma membrane and involves the hairpin-like tail at the C-terminal. Additionally, pediocin is thought to exhibit antimicrobial activity by interacting with the mannose phosphotransferase system [1]. In the case of the Ser13 → Cys13 conversion in Mut 1, it is predicted that the Cys amino acid tends to reside within the helical region, potentially disrupting the stabilization of the β-sheet region. Analysis of Mut 1 using DSSP data indicated an increase in the stabilization of the β-sheet region at 298 K (25 °C) compared to all other peptides. However, this mutation led to a substantial disruption in the stabilization of the β-sheet structure at various temperatures, resulting in the formation of random coils. Given the pivotal role of the β-sheet structure in integration, it was also observed that Mut 4 was the mutant most inclined to maintain overall structural stability, including the β-sheet region, particularly at elevated temperatures.

**Fig. 8:**
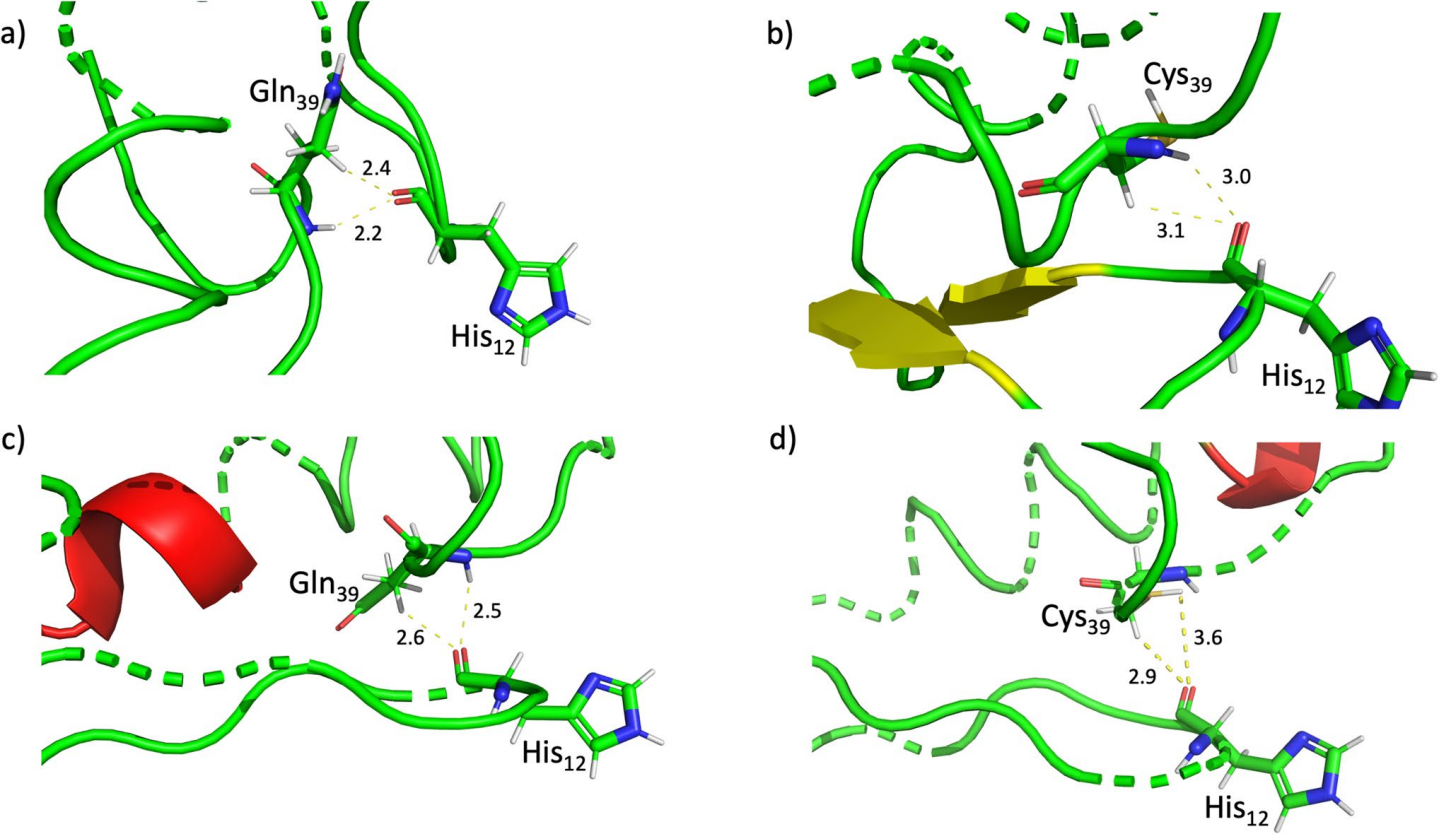
Change of hydrogen bond length and stabilization of peptide structure suggested by Bédar et al. [10] at 298 K (25 °C). **a** wild-type pediocin PA-1 (PDB code: 5UKZ), **b** Mut 1, **c** Mut 4, and **d** Mut 5

Bedárd et al. [10] suggested that the stabilization of the second disulfide bond (Cys24–-Cys44) and its potential to increase heat resistance may be due to the powerful 2.4 Å hydrogen bond between His12-Gln39. It was observed that, at 298 K (25 °C), mutations generally led to an increase in the bond length between these two residues. Notably, in the case of Mut 4 (Fig. 8c), these mutations contributed to preserving the helical structure and enhancing the stability of the mutant peptide at room temperature. As for Mut 1, the expansion of the bond between these two amino acids to 3.1 Å is believed to aid in safeguarding the β-sheet structure, which is believed to be crucial for cellular integration and antimicrobial activity at this temperature (Fig. 8b).

When evaluating disulfide bonds based on the Cα-Cα bond length, which is known to be between 3.5 Å and 7.5 Å [57], the literature suggests that the Cβ-Cβ bond length between two Cys residues is approximately 5.5 Å, and the S–S bonds average 2.04 Å in length [58]. One of the study’s aims was to investigate the potential formation of a third disulfide bond, but unfortunately, in silico simulations did not support the possibility of forming a third disulfide bond for pediocin and its mutant molecules under any conditions. At 313 K (40 °C), there was no significant difference in the lengths of disulfide bonds between the reference structure and mutant peptides. This suggests that the mutants could remain stable and exhibit antimicrobial activity at least to the same extent as the reference peptide. However, at 323 K (50 °C), Mut 1 lost its second disulfide bond during the simulation, indicating low structural stabilization. This raises doubts about its ability to display antimicrobial activity. At 373 K (100 °C), the original molecule, Pediocin PA-1, also lost its second disulfide bond, suggesting reduced stability compared to other mutants. Therefore, its antimicrobial activity at this temperature should be examined in vitro. On the other hand, Mut 5 lost its secondary disulfide bond at 394 K (121 °C), indicating that it is unlikely to exhibit antimicrobial activity at this high temperature. In contrast, Mut 4 did not lose its secondary disulfide bond at any of the temperatures tested. The discovery that Pediocin PA-1, Mut 1, and Mut 5 lose their disulfide bonds at this temperature challenges the common belief that pediocin retains activity at 100 °C and 121 °C [44, 55] during in silico simulations. This disparity suggests that bacteriocins expected to be effective at these temperatures could have applications in food safety processes for manufacturing foods treated at high temperatures in the industry. While Mut 4 shows encouraging results at these temperatures, it seems that the structural stability of the other mutant peptides created does not hold under similar conditions. Given the role of the second disulfide bond in antimicrobial activity, it is estimated that Mut 4, which maintains its disulfide bond stability at various temperatures in MD simulations, may also exhibit antimicrobial activity in vitro conditions.

Upon thorough analysis in this study, it became apparent that apart from the recognized disulfide bonds (Cys9-Cys14 and Cys24-Cys44), the structural stability of mutant peptides, crafted by the manual substitution of Cys residues, closely resembled that of the reference peptide. Of the five mutants developed, Mut 4 notably retained a stable structure within temperature ranges conducive to biological activity, the highest temperatures for pediocin production by relevant strains [12, 59], and industrial application temperatures. The thermostability of the commonly used nisin antimicrobial peptide endures up to 77 °C but diminishes beyond this point [60]. Given capacity of Mut4 to sustain its structure at elevated temperatures in MD simulations and the efficacy of pediocin at high temperatures [44, 55, 60], it emerges as a promising alternative for applications within the food industry. Particularly at industrially significant high temperatures studied herein, Mut 4 effectively preserved its β-sheet structure, ensuring structural stability. This facilitated the maintenance of a partially helical structure observed in MD simulations, significantly restricting fluctuations within the helical region at the amino acid level. Beyond the reference structure, it is recommended to observe the temperature-dependent structural stabilization, especially the anti-listerial effect of Mut 4 after MD simulation, through in vitro and in vivo studies. Despite the incapability to form a third disulfide bond, observed across all mutants, the existence of unbound free Cys residues, recognized for their role in enhancing structural stability [61], indicates that specifically synthesizing Mut 4 in vitro and validating the obtained data with temperature experiments and antimicrobial trials might yield significant insights. While these mutants lacked the ability to generate a third disulfide bond, suggesting potential limitations in serving as a more stable alternative to nisin, especially in high-temperature product manufacturing (e.g., industrial sterilization processes), investigating potential of Mut 4 through in vitro and experimental studies could reveal promising aspects related to its structural stability and antimicrobial effects.

## Conclusion

Pediocin and pediocin-like bacteriocins are peptides with promising industrial applications, particularly in terms of heat stability. However, literature on the direct impact of pediocin on the thermal stability of its peptide structure is limited. In this study, in silico simulations were conducted to reveal the thermal stabilization and structural changes of pediocin PA-1 at various temperatures, as well as the thermal stabilization and structural changes of new mutant pediocin structures created through Cys substitutions. Pediocin and pediocin-like bacteriocins are characterized by two disulfide bonds, with the second bond known to play a crucial role in antimicrobial activity. In MD simulations, the stability of the structure of peptides with double Cys mutations varied significantly. It is interesting to note that the addition of Cys mutations did not result in the formation of a third disulfide bond in any of the structures, even though the study aimed to investigate this possibility. Additionally, it was observed that the reference pediocin PA-1 structure could lose its secondary disulfide structure at high temperatures (above 100 °C), which raises questions about its stability under such conditions. The mutant peptide named Mut 4, on the other hand, demonstrated superior thermal stability, maintaining its disulfide bond structure even at high temperatures. This suggests that Mut 4 may have potential applications in industries that require the stability of antimicrobial peptides at elevated temperatures. It is crucial to underline that these findings are derived solely from in silico simulations, warranting further in vitro experiments to validate the results and evaluate the practical feasibility of Mut 4 in real-world scenarios. One limitation of this study is its reliance solely on predictive models obtained from MD simulations without support from in vitro experiments. Moreover, both in silico and in vitro demonstrations of the interactions between the generated mutants and the Man-PTS system receptor, a key player in the mechanistic antimicrobial activity of pediocin, could corroborate our assertions by illustrating the role of structural stabilization in the antimicrobial mechanism, which this study currently lacks. If substantiated by in vitro data, this study implies that Mut 4 could potentially serve as a more robust version of pediocin for industrial use, offering significant implications across various sectors, notably in the food industry. Overall, this research presents a promising path for developing thermally stable antimicrobial peptides, underscoring the significance of integrating computational simulations with experimental validation to advance the comprehension and application of such peptides.

## Data Availability

The datasets generated during and/or analyzed during the current study are available from the corresponding author on reasonable request.
